# Optimising White Wheat Bread Fortification with Vitamin D_3_ and Dietary Fibre: Balancing Nutritional Enhancement and Technological Quality

**DOI:** 10.3390/foods14122055

**Published:** 2025-06-11

**Authors:** Sabrina Boudrag, Elke K. Arendt, Celia Segura Godoy, Aylin W. Sahin, Laura Nyhan, Kevin D. Cashman, Emanuele Zannini

**Affiliations:** 1 School of Food and Nutritional Sciences, University College Cork, College Road, T12 YN60 Cork, Ireland; 124100939@umail.ucc.ie (S.B.); cseguragodoy@ucc.ie (C.S.G.); aylin.sahin@ucc.ie (A.W.S.); lnyhan@ucc.ie (L.N.); k.cashman@ucc.ie (K.D.C.); e.zannini@ucc.ie (E.Z.); 2 APC Microbiome Ireland, Biosciences Building, University College Cork, T12 YT20 Cork, Ireland; 3 Cork Centre for Vitamin D and Nutrition Research, School of Food and Nutritional Sciences, University College Cork, T12 YN60 Cork, Ireland; 4 Department of Medicine, University College Cork, T12 EC8P Cork, Ireland; 5 Department of Environmental Biology, Sapienza University of Rome, Piazzale Aldo Moro 5, 00185 Rome, Italy

**Keywords:** vitamin D deficiency, vitamin D_3_, dietary fibres, bread fortification, wholemeal, response surface methodology

## Abstract

Inadequate vitamin D and dietary fibre intake are growing public health concerns in Western countries, especially in regions with limited sunlight and diets rich in processed foods. Bakery products, widely consumed, offer a promising opportunity for nutritional fortification. This study explored the possibility of fortifying white wheat bread—a staple food but low in fibre—with vitamin D_3_ and various dietary fibres (oat fibre, pectin, cellulose, and beta-glucan). The goal was to enhance its nutritional profile while maintaining desirable bread qualities. Using Response Surface Methodology (RSM), an empirical model, optimised the fibre combination. A range of dough and bread analyses were conducted—including assessments of gluten structure, starch pasting, fermentation activity, crumb hardness, specific volume, and colourimetry. The results showed fibre addition weakened the gluten network and altered starch properties (reduced peak, final and breakdown viscosities)—reducing loaf volume (4.2 ± 0.4 mL/g vs. 4.8 ± 0.1 mL/g for the control)—though to a lesser extent than in wholemeal bread (2.4 ± 0.1 mL/g), while vitamin D_3_ inclusion had a minimal impact (4.0 ± 0.4 mL/g for white bread, 2.1 ± 0.0 mL/g for wholemeal bread). The study identified an optimal mix of soluble and insoluble fibres with vitamin D_3_ that preserved the texture, crumb structure, and appearance of standard white bread. The final product offered fibre levels (Total Dietary Fibre, TDF = 10.72 ± 0.31 g/100 g bread, vs. 3.81 ± 0.06 g/100 g for the control) comparable to those of wholemeal bread (TDF = 9.54 ± 0.67 g/100 g), with improved texture and volume. This approach presents an effective strategy to enhance staple foods, potentially improving public health through better nutrient intake without compromising consumer acceptance.

## 1. Introduction

Vitamin D is a fat-soluble secosteroid essential for calcium homeostasis and the maintenance of bone integrity, with emerging evidence suggesting its involvement in broader metabolic pathways [1]. It exists in two primary forms: these are vitamin D_2_ (ergocalciferol), synthesised in fungi and a very limited number of plants through ultraviolet B (UVB) radiation, and vitamin D_3_ (cholecalciferol), produced endogenously in human and animal skin upon UVB exposure. Populations residing at higher latitudes, particularly those experiencing prolonged periods of insufficient UVB radiation—referred to as “vitamin D winter”—are at heightened risk of vitamin D deficiency [2]. Epidemiological data indicate that approximately 40% of Europeans exhibit inadequate serum 25-hydroxyvitamin D [25(OH)D] levels, with 13% classified as deficient [3]. Consequently, there is a critical need to develop effective dietary strategies to address this public health concern.

Vitamin D_3_ is naturally present in limited food sources, such as fatty fish, liver, and egg yolks, while vitamin D_2_ is mainly found in certain mushrooms that have had UV exposure. However, these foods are not consistently consumed in sufficient quantities to meet daily requirements [2]. The European Food Safety Authority (EFSA) has established dietary reference values (DRVs) for vitamin D at 15 μg/day for individuals aged 1–17 years, adults, and the elderly, and at 10 μg/day for infants aged 7–11 months [4]. The relative efficacy of vitamin D_2_ versus D_3_ in elevating serum 25(OH)D levels remains debated. While some studies suggest that vitamin D_3_ exhibits superior efficacy in terms of raising 25(OH)D levels compared to vitamin D_2_, others report comparable efficacy between the two forms [5]. Data from systematic reviews suggests that the relative potency of vitamin D_2_ in terms of increasing serum 25(OH)D is lower than that of vitamin D_3_ [6,7].

Food fortification represents a viable strategy to enhance vitamin D intake, with commonly fortified vehicles including milk, orange juice, cereals, and mushrooms [2]. However, socioeconomic factors and dietary restrictions may limit access to fortified foods for certain populations. Bread, a widely consumed and cost-effective staple food, has been identified as a promising candidate for vitamin D fortification [5]. Bread is a carbohydrate-rich food, with around 50% of its energy derived from digestible carbohydrates, and it contributes an estimated 14–18% of daily energy intake in many developed countries [8]. Additionally, it is low-fat, serves as a source of dietary fibre (about 3 g/100 g bread), provides protein—accounting for roughly 12% of its total energy content—as well as minerals in lower proportions [9]. According to a report by the Association Internationale de la Boulangerie Industrielle (AIBI), the average bread consumption in Europe in 2013 was approximately 59 kg per person per year, which corresponds to about 160 g—or roughly four servings—per person per day [10]. In many regions, refined white wheat bread is preferred over wholemeal bread due to its palatability and texture [11,12]. However, white wheat flour is inherently low in dietary fibre, prompting interest in enhancing its nutritional profile through fibre enrichment. Indeed, despite its nutritional significance, the health image of white wheat bread has shifted in recent years due to concerns about a high glycaemic index, low micronutrient density, and reduced dietary fibre content [13,14,15]. This has driven innovation in bread formulation, including fortification with vitamins, minerals, fibre, and functional ingredients, to enhance its nutritional profile [16,17,18]. Incorporating dietary fibres and micronutrients like vitamin D is part of this trend, aiming to improve both health outcomes and product functionality. Dietary fibres are well-documented for their beneficial effects on colonic function, cholesterol metabolism, and glycaemic control [19]. Despite these benefits, global fibre intake remains suboptimal. In Europe, several studies estimate that more than 80% of adults fail to meet the recommended daily intake [11,12,16], which the World Health Organization (WHO) sets at >25 g per day to support overall health. Vitamin D and dietary fibres are considered shortfall nutrients of public health concern in the United States [20], suggesting that many individuals in the population consume intakes below the recommended levels.

This study investigated the dual fortification of white wheat bread with vitamin D_3_ and dietary fibres to address vitamin D deficiency and promote an adequate fibre intake. While previous research has highlighted the adverse effects of dietary fibres on dough rheology and bread quality—such as reduced loaf volume and altered crumb texture [21,22]—these effects are highly dependent on the type and combination of fibres used [23]. The synergistic effects of vitamin D_3_ and dietary fibres on bread characteristics remain underexplored, underscoring the novelty of this research. Most studies to date have focused on the health benefits of vitamin D_3_-fortified bread, particularly its role in increasing serum 25(OH)D levels, with, to our knowledge, no attention given to its potential impact on the technological or quality attributes of dough and bread. In this study, we aimed to assess not only the nutritional value of combining dietary fibre and vitamin D_3_, but also whether their inclusion—and potential synergy—could influence bread quality characteristics. A blend of soluble (pectin and beta-glucan) and insoluble (cellulose and oat fibre) dietary fibres was selected based on their complementary health benefits. Pectin, a soluble high-molecular-weight fermentable fibre, has demonstrated efficacy in modulating glucose and cholesterol levels while enhancing satiety [24]. Similarly, beta-glucan, another soluble fermentable fibre, is associated with improved lipid profiles and glycaemic control, as well as cardiovascular risk reduction [25]. Cellulose, an insoluble non-fermentable fibre, is recognised for its role in increasing faecal bulk and promoting bowel regularity due to its high water-holding capacity [26]. Oat fibre, comprising both insoluble cellulose (predominantly) and soluble beta-glucan, contributes to gut motility and cholesterol reduction while delaying gastric emptying and enhancing satiety [27,28].

A Design of Experiment (DoE) was implemented to evaluate the individual and combined effects of these fibres on bread properties, allowing us to develop an optimised high-fibre white wheat bread formulation. The aim was to achieve a fibre content comparable to wholemeal bread while maintaining the desirable techno-functional properties of white wheat bread. Wholemeal flour, despite its high-fibre content, often yields bread with inferior textural attributes, such as low specific volume and increased crumb hardness [29]. The optimised formulation sought to replicate standard white wheat bread’s sensory and functional qualities while incorporating a blend of fibres with demonstrated health benefits. Following the establishment of a fibre-optimised white wheat bread, vitamin D_3_ was incorporated to develop a product dually fortified with dietary fibre and vitamin D_3_.

Four experimental formulations were evaluated in comparison to a control white wheat bread: (1) fibre-fortified white wheat bread, (2) vitamin D_3_ + fibre-fortified white wheat bread, (3) wholemeal bread, and (4) vitamin D_3_-fortified wholemeal bread. Wholemeal flour served as a benchmark for fibre content, given its naturally high dietary fibre concentration. This study provides valuable insights into the potential of dual fortification strategies to address micronutrient deficiencies and improve dietary fibre intake, thereby contributing to public health nutrition.

## 2. Materials and Methods

### 2.1. Raw Ingredients

Refined white wheat baker’s flour (BF)—0.54 ± 0.04% ash—and coarse wholemeal flour (WMF)—1.44 ± 0.10% ash—were supplied by Odlums Group (Dublin, Ireland). According to official guidelines, wholemeal flours must contain all parts of the wheat kernel, including the bran, endosperm, and germ [30]. The fibre ingredients used for the optimised recipes were VITACEL^®^ L 600-30 cellulose from citrus peel (JRS, Rosenberg, Germany)—96.5% fibre; VITACEL^®^ Oat Fibre HF 401 (JRS, Germany)—86.0% fibre; GENU^®^ Pectin type BIG (CP Kelco, Atlanta, GA, USA) standardised with sucrose—45.2% fibre; and PromOat^®^ Beta-Glucan from oat grain (Lantmännen Oats AB, Stockholm, Sweden)—34 ± 2% beta-glucan and 2.0 g total sugars. Vital gluten (Roquette, Lestrem, France) was incorporated into the dietary fibre-fortified white wheat formulations to compensate for protein losses resulting from white flour replacement. Dry vitamin D_3_ 100 CWS/AM was supplied by DSM (Basel, Switzerland), consisting of 2.5 mg vitamin D_3_/g containing medium chain triglycerides, corn starch, acacia gum, sucrose, and α-tocopherol. Dry vitamin D_3_ was first manually blended with incremental amounts of BF or WMF in a small barrel drum to achieve a final concentration of 250 µg vitamin D_3_/100 g flour. This premix was then incorporated into BF or WMF to attain a final concentration in the bread between 15 and 20 μg of vitamin D_3_/100 g of bread, while accounting for baking losses and vitamin D_3_ degradation during the baking process. The target level of vitamin D_3_ in the bread was based on its application in a randomised controlled trial aimed at preventing vitamin D deficiency in older adults [31]. Instant active dried baker’s yeast (Bruggeman Brown Yeast, Puratos, Groot-Bijgaarden, Belgium), sugar (Siúcra, Dublin, Ireland), salt (Glacia British Salt Limited, Cheshire, UK), sunflower oil (Musgraves, Cork, Ireland) and tap water at 25 °C were also used for baking.

### 2.2. Design of Experiment (DoE) and Response Surface Methodology (RSM)

The Design of Experiment (DoE) was conducted using the Design-Expert 9 software (Stat-Ease, Inc., Minneapolis, MN, USA) following a central composite design and a quadratic design model, with four repetitions of the centre point. Refined white wheat flour (BF) was replaced with dietary fibres: oat fibre (from 0 to 30%, on a flour + fibre basis), cellulose, pectin, and beta-glucan (each added from 0 to 10%, on a flour + fibre basis) (Table 1).

These substitution levels were determined through preliminary experiments, aiming to incorporate the highest possible amounts of each fibre type without significantly affecting bread quality. The recipes followed the same proportions of sugar (2%), yeast (2%), salt (1.2%), and oil (3.2%) on a flour and fibre basis. Although most formulations included combinations of dietary fibres, the DoE included specific runs (i.e., runs 3, 15, 19, and 26) that represented the use of individual fibre types. These were beta-glucan, pectin, cellulose, and oat fibre, respectively. These runs enabled the evaluation of the individual effect of each fibre on specific volume and crumb hardness. The fibre content was calculated based on the known composition of the individual ingredients. The Response Surface Methodology (RSM) was used to build an empirical model for optimising the fibre fortification of a white wheat bread recipe using the DoE-generated recipes. It evaluated the effect of the four dietary fibres on sixteen responses related to the bread quality attributes, with the most relevant ones being the specific volume (mg/mL), crumb hardness (N), and the fibre content (g/100 g bread).

### 2.3. Water Content Optimisation

A Farinograph-TS^®^ (Brabender GmbH and Co KG, Duisburg, Germany) was used to optimise the water content of the different formulations, following the AACC International Standard Method 54-21. 300 g of flour ingredients (14% moisture basis) were blended for a minute in the mixing chamber to ensure homogeneity before water addition. Water was automatically injected to achieve a target dough consistency of 500 ± 20 Farinograph Units (FU), with the mixing chamber temperature set to 30 °C. Measurements were performed in triplicate.

### 2.4. Dietary Fibre Quantification

The Total Dietary Fibre (TDF), Insoluble Dietary Fibre (IDF) and Soluble Dietary Fibre (SDF) contents were measured in triplicate using the Rapid Integrated Total Dietary Fibre Assay Kit (K-RINTDF) (Megazyme, Bray, Ireland) following the AOAC Method 2022.01. from [32] with slight modifications as per Sahin et al. (2023) [33].

### 2.5. Vitamin D_3_ Content in Bread

Vitamin D_3_ concentration in bread was determined externally following an accredited liquid chromatography–mass spectrometry-based method reported as MP 1570 REV 3 2021 (Mérieux NutriSciences; Chelab Srl, Resana, Italy).

### 2.6. Dough Characteristics

All the experiments were carried out in triplicate.

#### 2.6.1. Dough Preparation

First, the dry ingredients (flour ingredients, salt and sugar) were premixed. Yeast was activated in the total amount of water at 25 °C for 10 min and was then added to the dry ingredients along with sunflower oil. The fibre-fortified recipes included dietary fibres and gluten (to compensate for protein loss). Vitamin D_3_-fortified BF or WMF were added to the corresponding recipes. Ingredients were mixed in a Kenwood Chef Classic mixer using a dough hook (Kenwood Manufacturing Co., Ltd., Havant, UK) at speed 1 for 1 min, after which the sides of the bowl were scraped down, followed by mixing at speed 2 for 7 min.

#### 2.6.2. Development of the Gluten Network

A Brabender GlutoPeak (GmbH and Co. KG, Duisburg, Germany) was used to evaluate the development of the gluten network, following the procedure previously described by Hoehnel et al. (2019) [34]. Then, 9 g of flour ingredients (based on 14% moisture) was dispersed in 36 °C deionised water, reaching a total weight of 18 g. The torque was measured over time at 36 °C and at a shear rate of 2750 rpm. The Peak Maximum Time (PMT) in seconds (s) and the maximum torque in Brabender Units (BU) were determined.

#### 2.6.3. Starch Pasting Properties

The starch pasting properties of the flour ingredients were measured using a Rapid Visco Analyzer (RVA) Super 3 (Newport Scientific, Warriewood, Australia), applying the Standard 1 pasting profile, according to the AACC method 76–21.02. Then, 3 g of flour ingredient (based on 14% moisture) was dispersed in deionised water to reach a final weight of 25 g. The peak viscosity (PV), breakdown viscosity (BV) and final viscosity (FV) in centipoise (cP) were determined.

#### 2.6.4. Dough Mixing and Pasting Properties

Dough mixing and pasting properties were determined using a Mixolab^®^ (Chopin, Villeneuve-la-Garenne CEDEX, France), according to the AACC International Method 54–60.01 following the standard “Chopin+” protocol. Flour ingredients (based on 14 % moisture) were loaded into the Mixolab^®^, and the water amount determined by the Farinograph-TS® was dispensed by the instrument to achieve a total dough weight of 75 g. Several parameters were determined during this process: DDT, the dough development time (min); C2 (Nm), protein weakening; C3 (Nm), starch gelatinisation; C4 (Nm), starch stability (α-amylase activity, shear-thinning); and C5 (Nm), starch retrogradation (during cooling).

#### 2.6.5. Dough Fermentation

Dough fermentation was monitored using a Rheofermentometer F3 (Chopin, Villeneuve-la-Garenne CEDEX, France). As previously described by Heitmann et al. (2015) [35], 300 g of dough was placed in a fermentation chamber with a 1500 g cylindrical weight on top. The chamber was sealed, and the dough was allowed to ferment for 3 h at 30 °C. During this process, the Height Maximum (HM) of the dough in millimetres (mm) and the volume of CO_2_ released during fermentation (mL) were measured.

#### 2.6.6. Breadmaking

After the dough preparation outlined above, 450 g of dough was placed in a greased tin (15 × 9.5 × 9.7 cm) and proofed for 90 min at 35 °C/75% humidity (KOMA SunRiser, Roermond, The Netherlands). The leavened dough was placed in a preheated deck oven (MIWE Condo, Arnstein, Germany), with a top/bottom temperature of 220 °C/230 °C. This was preceded by a 400 mL steam injection. The baking time was 35 min. The loaf was then cooled at room temperature for two hours before analysis.

### 2.7. Bread Quality Analysis

Specific volume and crust colour determination were performed on uncut loaves. For the other analyses, one loaf was cut into five slices of 25 mm thickness, with the ends omitted.

All the experiments were carried out in triplicate.

#### 2.7.1. Specific Volume

The specific volume of the bread loaves (mL/g) was measured using a Volscan Profiler (Stable Micro Systems, Surrey, UK), as previously reported by Bojňanská et al. (2021) [36].

#### 2.7.2. Crumb Structure

A C-Cell Imaging System (Calibre Control International Ltd., Warrington, UK) was used to measure the number of crumb cells and their diameter. The procedure follows the standardised protocol provided by the C-Cell Bread Imaging System manufacturer.

#### 2.7.3. Colour

The surface colours of the crust and crumb were determined using a Minolta Chroma Meter CR-331 (Konica Minolta Holdings Inc., Osaka, Japan) and the CIE L*a*b* system (Commission Internationale de l’Éclairage), as described by Angioloni and Collar (2009) [37]. The crust colour of the uncut loaf was measured ten times, after which the crumb colour was measured five times for each slice of bread. The colour difference (ΔE) between the samples and the white wheat bread control was calculated using the following equation:

(1) $$\Delta E=\sqrt{{\left(\Delta L^{*}\right)}^{2}+{\left(\Delta a^{*}\right)}^{2}+{\left(\Delta b^{*}\right)}^{2}}$$

where ΔL* = L*_control_ − L*_sample_, Δa* = a*_control_ − a*_sample_ and Δb* = b*_control_ − b*_sample_.

#### 2.7.4. Textural Properties

Texture analysis was performed on the breadcrumb using a TA-XT2i Texture Analyzer (Stable MicroSystems, Surrey, UK). A two-compression test was conducted on 25 mm thickness bread slices, using a 35 mm cylindrical probe with a strain of 40%, 1 mm/s pre-test speed, 5 mm/s test speed, and 10 mm/s post-test speed (5 s waiting time between the two compressions). The hardness of the breadcrumb was then assessed.

#### 2.7.5. Water Activity

The water activity of the crumb was measured at room temperature using an Aqua Laboratory Series 3 water activity meter (Decagon devices, Pullman, WA, USA).

#### 2.7.6. Staling Rate

The rate of staling was determined by measuring the difference in the hardness before and after five days of storage. Two loaves were prepared on day 0. One loaf was cooled for two hours and sliced, and the crumb hardness was measured. The other loaf was cooled and stored in a sealed bag for five days at room temperature. It was then sliced and measured for hardness. 

The staling rate was calculated as follows:

(2) $$Staling\;rate\;(\%)=\frac{Crumb\;hardness\;at\;day\;5-Crumb\;hardness\;at\;day\;0}{Crumb\;hardness\;at\;day\;0}$$

### 2.8. Statistical Analysis

Statistical analysis was performed using OriginPro 2024b (OriginLab Corporation, Northampton, MA, USA). The significance level was established at *p* value < 0.05. One-way ANOVA, Kruskal–Wallis testing (when the normality hypothesis is rejected), or Welch ANOVA (when the homogeneity of variance hypothesis is rejected) tests were carried out to determine the influence of dietary fibres and vitamin D_3_ fortification on the dough and bread characteristics. They were followed by pairwise comparison (*p* < 0.05) to determine significant differences between recipes.

## 3. Results

### 3.1. Experimental Design

**Figure 1:**
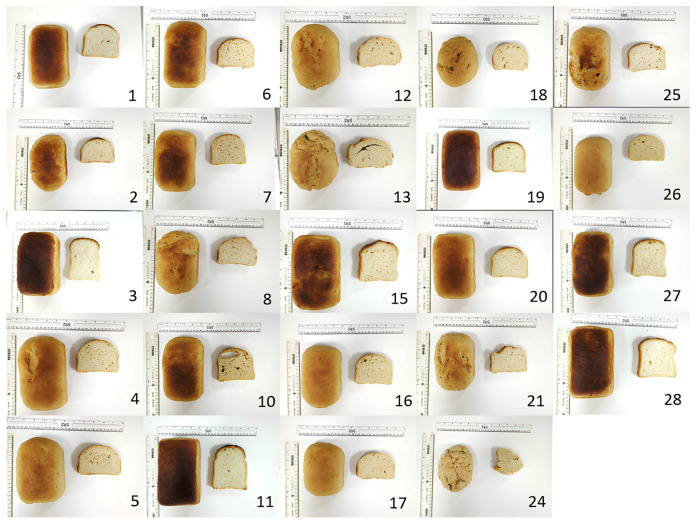
Bread loaves obtained according to Design of Experiment (DoE) recipes. Run 7 is shown as representation of four centre points. Photo of run 14 is missing.

**Figure A1:**
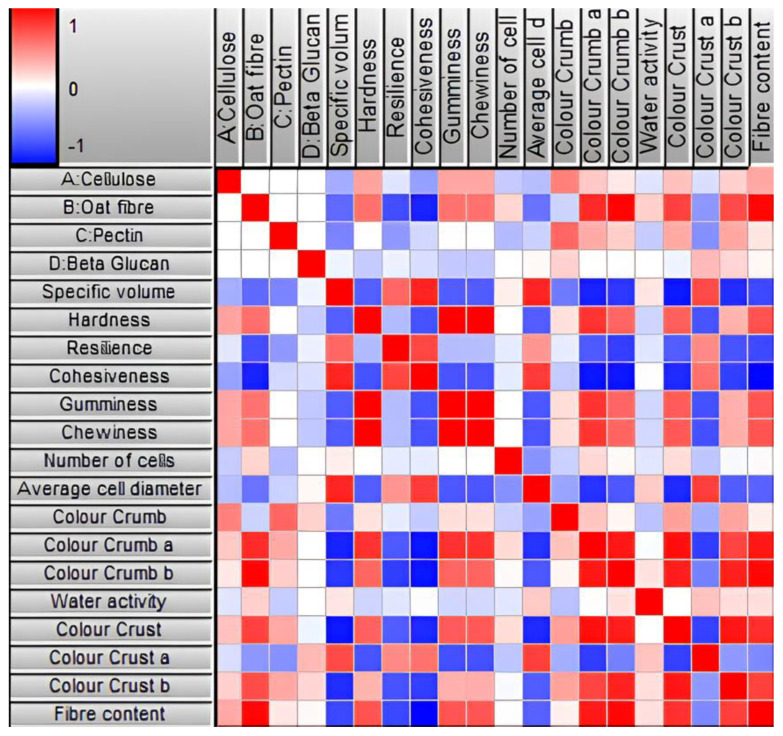
Correlation of fibre addition and bread quality characteristics in form of heat map obtained following the Design of Experiment (significance level α = 0.05). Red indicates positive correlation; blue indicates negative correlation.

Twenty-eight recipes were generated from the DoE, with a proportion of pectin, cellulose, and beta-glucan varying from 0 to 10%, and a percentage of oat fibre ranging from 0 to 30% (on a flour + fibre basis). The results of the DoE are displayed Figure A1 (see Appendix A) in a heat map representing the correlation between the type of dietary fibre added and the bread quality characteristics. The twenty-eight bread loaves, shown Figure 1, were then produced, and their attributes, corresponding to the sixteen responses entered in the DoE, were experimentally measured in duplicate. We input the data obtained for the three main responses (Table 2) into the design and processed it for the RSM analysis. The ideal parameters to enhance the quality of fibre-enriched white wheat bread were determined using a desirability-based multiple-response approach. This pre-optimisation technique integrates preferences and priorities for each variable involved. It was defined by a minimised hardness, a specific volume ranging between 3.8 and 4.9 mg/mL, and a fibre content between 10 and 15 g/100 g of bread. The target range for specific volume (3.8–4.9 mL/g) was determined based on typical values reported for white bread [38]. Specific volumes below 3.8 mL/g are indicative of inadequate leavening or a dense crumb structure, whereas values exceeding 4.9 mL/g may reflect over-aeration or structural instability. Additionally, 4.9 mL/g represents the upper limit permissible by the baking equipment due to oven height constraints. Crumb hardness was set to be minimised, as lower hardness generally indicates improved textural quality and consumer acceptability [39,40]. The target fibre content range of 10–15 g/100 g of bread was selected to ensure meaningful nutritional enhancement while maintaining product feasibility. This range corresponds to typical fibre levels in wholemeal bread, aligning with the nutritional profile intended for the final product [41,42]. Ten pre-optimised fibre-enriched recipes were generated (Table A1, see Appendix A), and of these, two formulations with the most desirable predicted characteristics—closest to the white control in terms of specific volume, crumb hardness, and fibre content, with 10–15 g/100 g bread—were selected for experimental analysis. The compositions of the two selected breads are presented in Table 3, with the corresponding analytical results shown in Table 4. Pre-optimised bread 1 was selected based on its more favourable characteristics. The composition of this bread is outlined in Table 5. Vitamin D_3_ was added to the recipe, targeting a concentration between 15 and 20 μg of vitamin D_3_/100 g of bread, to align with the EFSA recommendations [4]. In the vitamin D_3_-fortified wholemeal bread, 22.5% of vitamin D_3_-fortified flour (on a flour + fibre basis) was incorporated into the dough as a premix, while in the vitamin D_3_ + fibre-fortified white wheat bread, 25.5% (on a flour + fibre basis) was added.

### 3.2. Dietary Fibre Determination

The TDF, IDF, and SDF contents of all the bread recipes are reported in Table 6. In the white wheat bread containing fibres and vitamin D_3_, the TDF content was 10.72 ± 0.31 g/100 g bread, comprising 78% IDF and 22% SDF. In comparison, the control contained 3.81 ± 0.06 g TDF/100 g bread, with 64% IDF and 36% SDF. The wholemeal bread contained 8.54 ± 0.24 g TDF/100 g bread, with 84% IDF, and 16% SDF. Hence, the TDF of the vitamin D_3_ + fibre-fortified white wheat bread was higher by almost 3-fold and 1.3-fold compared to the white wheat bread control and the wholemeal bread, respectively. The IDF content in the vitamin D_3_ + fibre-fortified white wheat bread was around 3.4 times higher compared to the control white bread and around 1.2 times higher compared to the wholemeal bread due to the addition of cellulose and oat fibre. The SDF content was almost doubled compared to both the white wheat bread control and the wholemeal bread as a result of the inclusion of beta-glucan and pectin.

### 3.3. Dough Properties Determination

**Figure 2:**
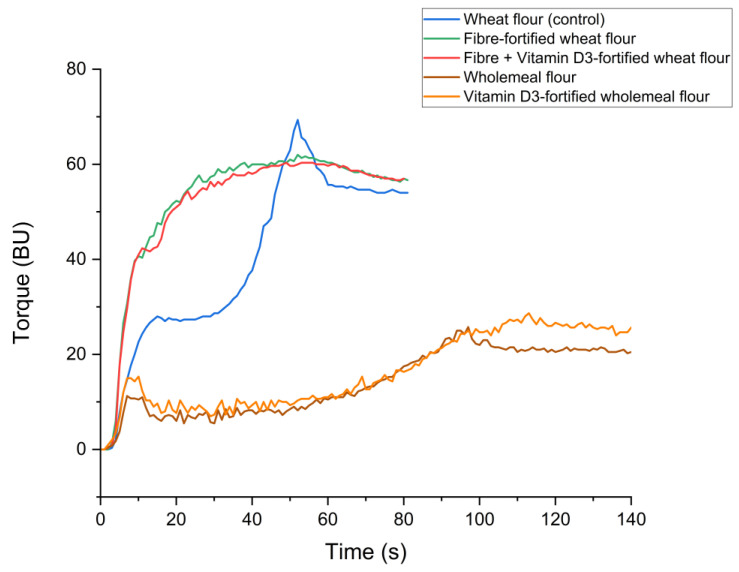
Gluten network development represented as torque (BU) profile over time (s) at 36 °C.

The maximum torque (BU) and the PMT (s) were determined by the GlutoPeak to assess the gluten network strength and the values are presented in Table 7, while the gluten network development curves are displayed Figure 2. The white wheat control showed the highest maximum torque at 69.3 ± 2.3 BU with a PMT of 53.0 ± 2.0 s. The gluten network development curve obtained for the control shows a typical pattern. (1) Initial phase: This involves the hydration of the gluten-forming proteins with weak interactions, leading to the beginning of gluten network development, which is shown by a small increase in torque and a plateau. (2) Aggregation phase: This comes as a result of the constant mixing and interactions between gluten proteins increase, leading to the formation of a strong network, characterised by an increase in torque until the peak maximum. (3) Breakdown phase: The continuous mixing breaks down the gluten network, causing a decrease in torque [43,44]. The gluten development curve profiles for the fibre-fortified and the vitamin D_3_ + fibre-fortified white wheat recipes showed similar trends and differed from the control, with no initial phase and a very rapid increase in torque leading to a plateau. The fibre-fortified white wheat formula did not show results significantly different to the white wheat control for the PMT but presented a significantly decreased maximum torque. Similar findings were reported by Sempio et al. (2024), who investigated the fortification of refined white wheat flour with a blend of arabinoxylan, resistant starch type IV, and cellulose [23]. They also observed a significant reduction in maximum torque for fibre-fortified white flour compared to standard white wheat flour. However, unlike our results, their fibre-fortified samples exhibited markedly lower PMT than the control, indicating different aggregation behaviour. This variation in curve profile may be attributed to the different combinations of fibres used in their study compared to ours. In a 2010 study, Goldstein et al. investigated the effects of cellulose addition on gluten using GlutoPeak analysis [45]. Similarly, they reported that cellulose led to a decrease in PMT and a rapid increase in torque, indicating the disruption and weakening of gluten aggregation. Fortification with both fibre and vitamin D_3_ also did not significantly affect the PMT but caused a significant decrease in the maximum torque compared to the control. Gluten network development was significantly different for the wholemeal recipes. The profile of the two gluten development curves showed an initial phase, similar to the white wheat control, rapidly leading to a low peak maximum, followed by a breakdown. The PMT for the wholemeal flour was 104.0 ± 12.5 s and the torque maximum was 33.7 ± 4.2 BU. These values were similar to those of the vitamin D_3_-fortified wholemeal. Comparable curve profiles were reported by Neylon et al. (2021), who applied GlutoPeak analysis to wholemeal flour and observed a similar maximum torque (29.0 ± 1.0 BU) and a PMT of 141.33 ± 15.18 s [46].

**Figure 3:**
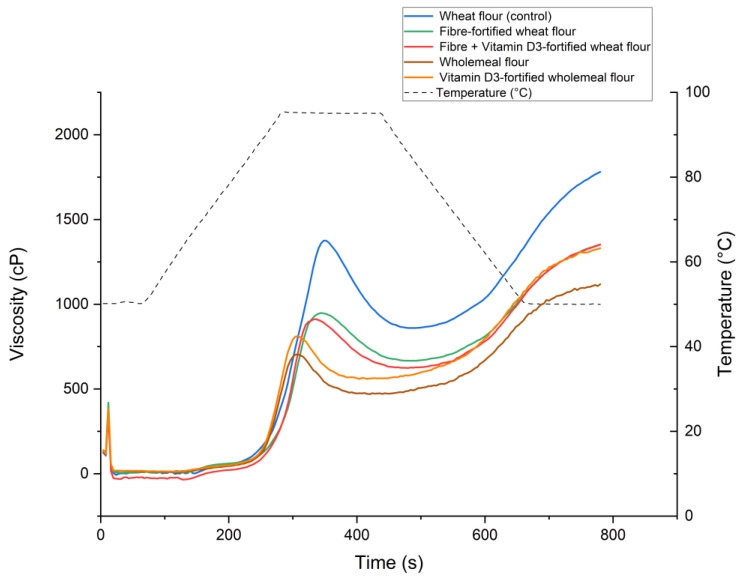
Starch pasting represented as viscosity (cP) profile over time (s).

The starch pasting properties of the flour blends were measured using the Rapid Visco Analyzer. PV, BV, FV in centipoise (cP), and the development curves were assessed and are shown, respectively, in Table 7 and Figure 3. The starch pasting properties of the fibre-fortified white wheat flour were significantly affected, with decreased PV, FV, and BV compared to the control. The addition of both vitamin D_3_ and dietary fibres in white flour led to a significant drop in the PV, FV, and BV compared to the white wheat flour control. Wholemeal flour showed the lowest starch pasting properties of all flours. PV, FV, and BV for the vitamin D_3_-fortified wholemeal flour were also significantly lower than for the white wheat control.

**Figure A2:**
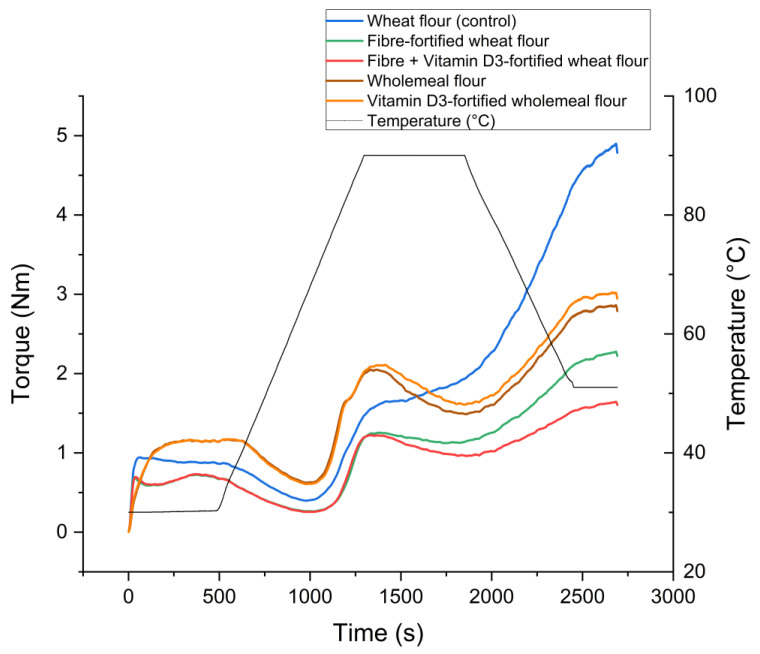
Mixolab torque curves (Nm) over time (s) obtained using the “Chopin+” protocol, illustrating dough behaviour under combined mechanical and thermal stress.

Mixolab^®^ analysis was conducted to obtain a general overview of the flour quality and its response to simultaneous thermal and mechanical stress. However, more detailed insights into specific aspects of dough functionality—gluten aggregation and starch pasting behaviour—were obtained using GlutoPeak and RVA, respectively. The DDT as well as the torques C2, C3, C4 and C5 were determined, and the results are shown in Table 7. The curves obtained are presented Figure A2 (see Appendix A). The addition of dietary fibres to white wheat flour significantly increased the DDT (6.1 ± 0.4 min) compared to the white wheat flour control (1.4 ± 0.8 min) and significantly decreased all torques. The incorporation of vitamin D_3_ + dietary fibres into white flour displayed results comparable to the fibre-fortified white wheat flour. Wholemeal flour showed a significantly higher DDT than the white wheat control (7.8 ± 1.9 min), as well as significantly different torques. The DDT for the vitamin D_3_-fortified wholemeal flour was 7.9 ± 2.0 min, with all torque values significantly different compared to the white wheat control.

A Rheofermentometer was used to assess the fermentation capacity of the dough, with the results illustrated in Table 7. HM (mm) and the total CO_2_ produced (mL) were determined for each recipe. The addition of dietary fibres in white wheat flour had a significant influence on fermentation as it reduced the HM (56.1 ± 2.7 mm) and resulted in an increase in the total CO_2_ produced (2151 ± 66.5 mL) compared to the control white wheat dough (HM = 65.9 ± 2.8 mm; total CO_2_ produced = 1958 ± 6.6 mL). The addition of vitamin D_3_ + fibres in white flour did not result in any significant changes in fermentation parameters compared to the control. The fermentation characteristics of the wholemeal formulations differed significantly from those of the white wheat recipes. The wholemeal dough exhibited an HM of 30.4 ± 0.8 mm and a total CO_2_ production of 1733 ± 38.6 mL. Previous studies also observed a significant reduction in the HM for wholemeal dough compared to white wheat dough [46,47]. The vitamin D_3_-fortified wholemeal dough showed similar results than the wholemeal control.

### 3.4. Bread Characteristics Analysis

**Figure 4:**
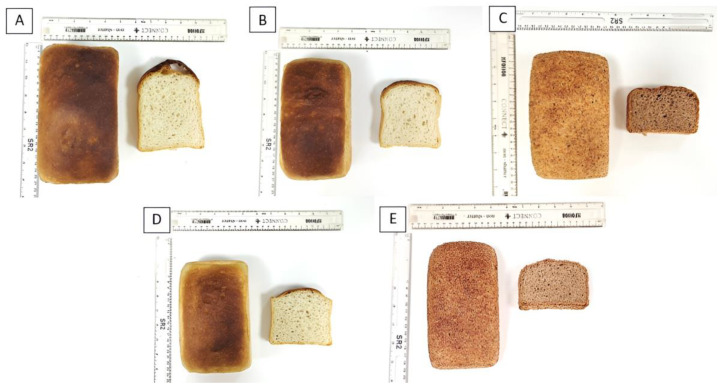
Baked loaves. (**A**) White wheat bread (control), (**B**) fibre-fortified white wheat bread, (**C**) wholemeal bread, (**D**) vitamin D_3_ + fibre-fortified white wheat bread, (**E**) vitamin D_3_-fortified wholemeal bread.

Figure 4 illustrates the appearance of the baked loaves.

As per Table 8, the measured vitamin D_3_ average values in the vitamin D_3_ + fibre-fortified white and the vitamin D_3_-fortified wholemeal breads (27.2 ± 7.4 and 28.4 ± 7.8 µg/100 g bread, respectively) were close to the target level of addition of 15–20 µg/100 g bread.

The specific volume of the bread loaves was measured with a Volscan Profiler and the results are presented Table 8. All recipes showed a significantly lower specific volume than the white wheat bread control (4.8 ± 0.1 mL/g), with the lowest specific volume observed for the wholemeal breads. Comparable specific volume values for wholemeal bread have been reported in previous studies [46,47].

The number of cells and their diameter were determined using a C-Cell Imaging System and the results are shown in Table 8. Fibre-fortified white wheat bread showed a significantly decreased cell diameter compared to the white wheat bread control, but no significant changes in the number of cells were noted. The incorporation of both vitamin D_3_ and fibres into white flour resulted in a significant reduction in cell diameter compared to the control, but there was no significant change in the number of cells. The structure of the wholemeal bread crumb differed significantly from the white wheat recipes, exhibiting a cell count of 3660 ± 194 and a cell diameter of 1.9 ± 0.1 mm. Previous research has reported comparable findings [46,47]. Similar values to those with the wholemeal bread were observed for the vitamin D3-fortified wholemeal bread.

The differences in crust (ΔE_crust_) and crumb (ΔE_crumb_) colour between the samples and the white wheat control bread were assessed using colourimetry analysis and expressed as ΔE (Table 8). The wholemeal recipes showed the greatest crumb and crust colour differences. No significant differences in crumb or crust colour were observed between the fibre-fortified white wheat and the vitamin-D_3_ + fibre-fortified white wheat bread.

The texture analysis of the bread loaves allowed the assessment of the crumb hardness, and the results are displayed Table 8. The wholemeal recipes showed the highest hardness of all samples, with hardness values of 29.9 ± 3.5 N and 23.5 ± 3.8 N determined for the vitamin D_3_-fortified wholemeal bread, and the wholemeal bread, respectively. Both were significantly higher than those observed for the white wheat bread control (2.3 ± 0.4 N). Similar results have been reported by Neylon et al. (2021) [46]. The hardness of the fibre-fortified white wheat bread and the vitamin D_3_ + fibre-fortified white wheat bread did not differ significantly from the control.

The results for the water activity measurements in the breadcrumb are shown Table 8. The water activity values ranged from 0.97 to 0.98, and no significant differences were observed between the samples.

The staling rate was measured for all bread recipes, as displayed in Table 8. The inclusion of fibre ingredients alone and the inclusion of vitamin D_3_ + fibre in white wheat flour did not result in a significant difference in the staling rate compared to the white wheat control (3.0 ± 1.0%). Wholemeal breads showed a significantly lower staling rate than the control.

## 4. Discussion

The experimental design yielded an optimised formulation for white wheat bread enriched with dietary fibres and vitamin D_3_. The final product contained approximately 10 g of Total Dietary Fibre (TDF) per 100 g of bread, comprising approximately 80% Insoluble Dietary Fibre (IDF) and 20% Soluble Dietary Fibre (SDF). This composition meets the criteria for a “high-fibre” claim as defined by Regulation (EC) No 1924/2006 of the European Parliament and of the Council (2006) [48], which requires at least 6 g of fibre per 100 g of product. Including dietary fibres significantly elevated IDF content compared to standard white wheat bread and enhanced SDF concentrations compared to standard white wheat bread and wholemeal bread. The primary dietary fibre in white wheat flour is arabinoxylan, accounting for 60–70% of TDF [49], followed by fructans and beta-glucan. Wholemeal flour is predominantly composed of IDF (65–80%, depending on the cultivar), with cellulose being the major component of wheat bran. SDF, present in smaller quantities, primarily consists of arabinoxylan and beta-glucan [26]. Arabinoxylan is well-documented for its prebiotic effects, antioxidant properties, the regulation of short-chain fatty acids, and beneficial impacts on postprandial blood glucose control and immune system modulation [50,51]. Incorporating oat fibre, cellulose, beta-glucan, and pectin, alongside naturally occurring arabinoxylan in wheat, resulted in a more diverse dietary fibre profile, enhancing the potential health benefits of the bread. IDF, such as cellulose and oat fibre, promotes gut motility, while high-molecular weight Soluble Dietary Fibre (SDFP, e.g., pectin and beta-glucan) and low-molecular weight Soluble Dietary Fibre (SDFS) play critical roles in glucose and cholesterol regulation. Vitamin D_3_ + fibre-fortified white wheat bread showed a significantly higher TDF than fibre-fortified white wheat bread. It is important to note that the AOAC Method of 2022.01., used to determine the dietary fibre content, has a repeatability relative standard deviation for TDF analyses < 3.60%, which may account for these differences [32].

The addition of dietary fibres significantly influenced the techno-functional properties of both the dough and bread. Increased fibre content has been shown to reduce loaf volume and increase crumb hardness [21]. The analyses of the 28 runs showed that insoluble fibres, such as cellulose and oat fibre, contributed to increased crumb hardness. In contrast, soluble fibres like beta-glucan maintained a specific volume and texture comparable to the control (Table 2). This can be attributed to the lack of direct interaction between beta-glucan and gluten proteins, as demonstrated by Zhou et al. (2021) [52]. Although individual fibre ingredients led to undesirable bread properties, their synergistic combination resulted in an optimised fibre-fortified white wheat bread recipe.

Vitamin D_3_ was incorporated into the formulation, revealing a 50% loss after baking. This aligns with findings by Mocanu et al. (2009), who reported a 40–50% loss of vitamin D_3_ in bread buns containing 125 μg (5000 IU) vitamin D_3_/100 g dough and baked at 260–270 °C for 15 min [53]. Lower losses (15–31%) have been observed in studies using smaller amounts of vitamin D_3_ (8–11 µg/100 g dough) [54]. Vitamin D_3_ degradation is influenced by different factors such as oxidation [30,31], as well as temperature and initial concentration, with higher temperatures and concentrations leading to greater degradation [55]. In the presence of heat, vitamin D_3_ undergoes a reversible isomerisation reaction, being converted into pre-vitamin D_3_ [56]. The presence of reactive oxygen species (ROS) can lead to the oxidation of vitamin D_3_, forming various degradation products. Lipid peroxidation, a process that generates ROS, may contribute to vitamin D_3_ degradation, particularly in fortified foods containing oils or fats [57,58]. A study by Tabibian et al. (2017), demonstrated that fermentation lasting over 60 min, as in the current study, regardless of temperature, led to a decrease in vitamin D_3_ concentration, likely due to oxidation [59]. Even accounting for loss during baking, a 40 g serving of this fortified bread (equating approximately to one medium slice) provides approximately 70% of the daily recommended vitamin D_3_ intake as well as around 16% of the daily recommended fibre intake. The final bread formulation in our study contained in excess of 20 µg (800 IU) of vitamin D_3_ per 100 g, qualifying it as “high in vitamin D” according to Council Directive 90/496/EEC (1990) [60]. This aligns with EFSA’s Tolerable Upper Intake Level (UL) for vitamin D, which are set at 50 and 100 µg (2000 and 4000 IU) per day for those aged 1–10 years and 11 years and older, respectively [61]. 

Including dietary fibres significantly influenced the mixing and pasting properties of both white wheat and wholemeal dough and bread. Fibre inclusion in white wheat flour, but not vitamin D_3_, disrupted gluten network development, leading to longer dough development times and weaker gluten networks compared to the control. This was evidenced by reduced maximum torque in GlutoPeak tests and lower C2 (protein weakening) torque values in Mixolab^®^ analysis. This disruption is attributed to competition for water between gluten proteins and fibres, as well as interactions between gluten proteins and fibres [52,62]. The GlutoPeak analysis revealed distinct torque profiles for the two white wheat fibre-fortified recipes, suggesting the existence of non-covalent binding between fibres and gluten proteins [52,63]. Additionally, soluble fibres increase dough liquor viscosity in the water-soluble phase of the dough, which may account for the observed curve profiles [64,65]. Soluble fibres also have a high water-holding capacity, which can potentially limit the hydration of the gluten network [66,67], possibly explaining our results. In the wholemeal recipes, coarse bran particles further weaken the gluten network by interfering with its development [68]. Starch pasting properties were unaffected by vitamin D_3_ addition but were significantly influenced by fibre inclusion. Lower peak viscosity (PV) values indicated reduced starch granule swelling and water absorption, while lower final viscosity (FV) values suggested reduced retrogradation, which can improve bread texture [69]. This may be due to a reduction in available starch in fibre-enriched flour or hindered starch gelatinisation, caused by fibres competing for water, which aligns with the reduced C3 torques (starch gelatinisation) determined by the Mixolab^®^ analysis [70]. Zhuang et al. (2024) proposed three mechanisms: competition for water, the encapsulation of starch granules by fibres, and the cross-linking of starch granules by fibres [71]. Wholemeal recipes exhibited significantly lower PV due to the higher water-binding capacity of bran particles, reducing the water available for starch hydration [68].

The inclusion of fibre in white wheat flour influenced fermentation dynamics, reducing maximum dough height while increasing CO_2_ production compared to the control. This effect may be attributed to the presence of fermentable sugars in the beta-glucan ingredient and the sucrose added to standardise the pectin content, which serve as substrates for yeast metabolism [72]. Otherwise, it can be attributed to the enzymatic breakdown of fibre into fermentable sugars by endogenous enzymes [73]. It is also important to acknowledge that commercial fibre sources may contain uncharacterised compounds that could affect fermentation outcomes. In the vitamin D_3_ + fibre-fortified white wheat formulation, vitamin D_3_ appeared to inhibit yeast CO_2_ production. This suppression may be because of vitamin D_3_ interfering with ergosterol incorporation into yeast membranes. Ergosterol, a fungal sterol structurally analogous to vitamin D, is essential for maintaining membrane integrity. Exogenous sterols such as vitamin D_3_ could compete with ergosterol, potentially disrupting membrane functionality and reducing fermentation efficiency [74].

Conversely, in the vitamin D_3_-fortified wholemeal formulation, no significant effect was observed on CO_2_ production, likely due to the limited availability of simple sugars—a more dominant factor in fermentation performance than vitamin D_3_. The reduced maximum dough height in fibre-fortified and vitamin D_3_ + fibre-fortified white wheat formulations can be explained by the fibre-induced disruption of the gluten network, impairing gas cell expansion [75,76]. While soluble fibres are known to increase dough liquor viscosity and delay gas cell coalescence [64,65], the predominance of insoluble fibres in this study may have counteracted these effects, leading to the observed results.

Fibre fortification significantly affected bread quality, impairing dough proofing and reducing specific volume. Wholemeal formulations exhibited particularly low specific volumes due to the physical and chemical effects of bran and germ, which restrict gas cell expansion. Bran particles act as physical barriers at gas cell interfaces, while germ-derived lipid-metabolising enzymes interfere with gas cell stabilisation—a process normally facilitated by lipid–gluten interactions at the gas–liquid interface [38,77,78].

Fibre inclusion also reduced gas cell diameter, further supporting the role of gluten network disruption in limiting expansion. Wholemeal loaves displayed fewer and smaller gas cells than white wheat loaves. Fibre-fortified white wheat recipes, in contrast, did not show any significant difference in the number of cells compared to the white wheat control. 

Texture analysis revealed increased crumb hardness in wholemeal bread loaves, likely due to their lower air incorporation and specific volume [79]. Fibre-fortified and vitamin D_3_ + fibre-fortified white wheat recipes, in contrast, showed no significant difference in hardness. Staling rates were comparable between fibre-fortified white wheat recipes and the control white wheat bread, contrasting with starch retrogradation behaviour, as the fibre-fortified white wheat formulations exhibited significantly lower final viscosity and C5 (starch retrogradation) torque values compared to the control. A study by Monteau et al. (2017) highlights the significant role of water losses—on par with starch retrogradation—in bread staling. This effect may be more pronounced in fibre-fortified white wheat formulations compared to the control, potentially explaining our observations [80]. Wholemeal formulations demonstrated slower staling, aligning with their lower final viscosity and lower C5 torques values. This was possibly due to enhanced crumb water retention [81].

Colour analysis indicated the existence of significant differences between fortified and control breads. While fibre-fortified white wheat recipes showed only minor crumb colour variation, wholemeal formulations exhibited pronounced ΔE values. Crust colour differences were evident across all recipes. 

Water activity remained unaffected by fibre or vitamin D_3_ inclusion. It was initially hypothesised that variations in formulation—especially those containing fibres—might alter water binding and distribution within the bread matrix, potentially impacting water activity, thus impacting shelf-life and stability.

## 5. Conclusions

This study identified an optimal formulation of Insoluble and Soluble Dietary Fibres combined with vitamin D_3_, yielding a fortified bread with properties comparable to conventional white wheat bread while complying with European regulations for “high-fibre” and “high-vitamin D” claims. The developed bread provides a dietary fibre content equivalent to that of wholemeal bread, while incorporating a blend of soluble and insoluble fibres, both of which are associated with distinct health benefits. Notably, it retains white wheat bread’s key visual and structural attributes, including colour, texture, and crumb structure. Further investigation into the mechanistic interactions between dietary fibres, vitamin D_3_ co-carriers (e.g., medium-chain triglycerides [MCTs], acacia gum), and dough macromolecules could help to elucidate the underlying microscopic processes influencing dough behaviour and nutrient stability. Additionally, novel baking technologies, such as Pulsed Electric Fields (PEFs), could be used to minimise vitamin D_3_ degradation during processing. PEFs have demonstrated efficacy in preserving heat-sensitive nutrients like vitamin C [82] and may offer similar advantages for vitamin D_3_ stability. The development of encapsulation systems for vitamin D_3_, as used in some dairy-based foods, represents another promising approach for bread [83]. Majeed and Rather (2024) reviewed a number of techniques which could prove effective strategies for enhancing the heat-stability and bioavailability of vitamin D depending on the application, such as nanoliposomes or electrospinning [84]. Given its favourable nutritional profile—rich in both dietary fibres and vitamin D_3_, with a 40 g serving providing ~16% of the daily recommended fibre intake and ~70% of the daily recommended vitamin D intake—this dually fortified bread represents a viable strategy for promoting a sustainable, affordable, and balanced diet. This advancement holds promise for addressing vitamin D deficiency through an accessible, safe, and health-promoting food product.

## Figures and Tables

**Table 1:** The twenty-eight dietary fibre combinations for white wheat flour fortification generated by the Design of Experiment (DoE). Percentages are expressed on a flour + fibre basis. Run 11 represents the white wheat bread control. Runs 7, 9, 22 and 23 correspond to the four repetitions of the centre point. Runs 3, 15, 19, and 26 represent the use of individual fibre types—beta-glucan, pectin, cellulose, and oat fibre, respectively. BF = baker’s flour (refined white wheat flour).

Run	BF (%)	Oat Fibre (%)	Cellulose (%)	Pectin (%)	Beta-Glucan (%)	Gluten (%)
1	80	0	10	0	10	2.9
2	80	0	0	10	10	2.9
3	90	0	0	0	10	1.2
4	65	15	10	5	5	5.7
5	50	30	10	0	10	8.0
6	65	15	5	5	10	5.5
7	70	15	5	5	5	4.9
8	70	0	10	10	10	4.6
9	70	15	5	5	5	4.9
10	60	30	0	0	10	6.3
11	100	0	0	0	0	0
12	50	30	0	10	10	8.0
13	50	30	10	10	0	8.6
14	75	15	0	5	5	4.0
15	90	0	0	10	0	1.7
16	55	30	5	5	5	7.4
17	60	30	0	10	0	6.9
18	40	30	10	10	10	9.7
19	90	0	10	0	0	1.7
20	75	15	5	0	5	4.0
21	65	15	5	10	5	5.7
22	70	15	5	5	5	4.9
23	70	15	5	5	5	4.9
24	60	30	10	0	0	6.9
25	80	0	10	10	0	3.4
26	70	30	0	0	0	5.1
27	75	15	5	5	0	4.3
28	85	0	5	5	5	2.3

**Table 2:** Specific volume (mL/g), hardness (N) and fibre content (g/100 g bread) for 28 recipes generated by the Design of Experiment (DoE), displayed as average values ± standard deviation.

Run	Specific Volume (mL/g)	Hardness (N)	Fibre Content (g/100 g)
1	3.10 ± 0.21	6.28 ± 1.44	10.97
2	2.33 ± 0.05	7.40 ± 0.76	8.06
3	4.93 ± 0.07	1.68 ± 0.25	6.55
4	2.66 ± 0.02	8.93 ± 0.81	18.34
5	2.21 ± 0.04	17.42 ± 2.50	24.01
6	2.82 ± 0.07	7.23 ± 2.44	16.45
7	3.00 ± 0.05	5.96 ± 0.84	15.90
8	1.94 ± 0.05	14.80 ± 1.98	13.06
9	3.00 ± 0.05	5.96 ± 0.84	15.90
10	2.96 ± 0.21	9.72 ± 1.17	19.68
11	4.91 ± 0.07	2.38 ± 0.27	4.90
12	1.62 ± 0.08	23.55 ± 4.65	21.27
13	1.31 ± 0.13	54.86 ± 4.99	26.23
14	3.52 ± 0.01	3.24 ± 0.78	13.66
15	2.72 ± 0.18	4.65 ± 1.59	6.96
16	2.38 ± 0.10	11.13 ± 1.06	22.36
17	1.76 ± 0.01	19.59 ± 2.59	20.15
18	1.33 ± 0.01	48.99 ± 3.74	26.07
19	3.70 ± 0.10	7.55 ± 0.85	10.59
20	2.92 ± 0.01	8.14 ± 1.34	15.15
21	1.99 ± 0.01	13.59 ± 0.92	16.68
22	3.00 ± 0.05	5.96 ± 0.84	15.90
23	3.00 ± 0.05	5.96 ± 0.84	15.90
24	1.27 ± 0.11	104.19 ± 15.02	25.39
25	2.32 ± 0.01	8.38 ± 1.03	11.54
26	2.27 ± 0.03	28.81 ± 2.95	19.68
27	4.10 ± 0.02	2.74 ± 0.79	15.59
28	4.47 ± 0.14	1.61 ± 0.27	9.90

**Table 3:** Formulations for two pre-optimised breads selected following Response Surface Methodology (RSM). % values are expressed on a flour + fibre basis. BF = baker’s flour (refined white wheat flour).

Sample	BF (%)	Oat Fibre (%)	Cellulose (%)	Pectin (%)	Beta-Glucan (%)	Gluten (%)
Pre-optimised-1	82.8	7.6	3.0	1.4	5.2	4.4
Pre-optimised-2	81.1	6.0	2.6	2.6	7.7	4.8

**Table 4:** Specific volume (mL/g), hardness (N) and fibre content (g/100 g bread) for two selected pre-optimised breads, displayed as average values ± standard deviation.

Sample	Specific Volume (mL/g)	Hardness (N)	Fibre Content (g/100 g)
Pre-optimised-1	4.17 ± 0.33	2.38 ± 0.49	10.86
Pre-optimised-2	4.39 ± 0.12	3.35 ± 0.91	10.61

**Table 5:** Bread recipes expressed in % of whole recipe (WR) or on flour + fibre basis (FF). BF = baker’s flour (refined white wheat flour), WMF = wholemeal flour.

Ingredients	White Wheat Bread (Control)		Fibre-Fortified White Wheat Bread		Vitamin D_3_ + Fibre- Fortified White Wheat Bread		Wholemeal Bread		Vitamin D_3_-Fortified Wholemeal Bread	
Flour Ingredients	% WR	% FF	% WR	% FF	% WR	% FF	% WR	% FF	% WR	% FF
Flour (BF or WMF)	58.1	100.0	42.3	82.8	29.3	57.3	58.9	100	45.7	77.5
Vitamin D_3_-fortified flour (250 μg VitD_3_/100 g BF or WMF)	-	-	-	-	13.0	25.5	-	-	13.3	22.5
Oat fibre	-	-	3.9	7.6	3.9	7.6	-	-	-	-
Beta-glucan	-	-	2.7	5.2	2.7	5.2	-	-	-	-
Cellulose	-	-	1.5	3.0	1.5	3.0	-	-	-	-
Pectin	-	-	0.7	1.4	0.7	1.4	-	-	-	-
Gluten	-	-	2.3	4.4	2.3	4.4	-	-	-	-
Salt	0.7	1.2	0.6	1.2	0.6	1.2	0.7	1.2	0.7	1.2
Sugar	1.2	2.0	1.0	2.0	1.0	2.0	1.2	2.0	1.2	2.0
Yeast	1.2	2.0	1.0	2.0	1.0	2.0	1.2	2.0	1.2	2.0
Sunflower oil	1.9	3.2	1.6	3.2	1.6	3.2	1.9	3.2	1.9	3.2
Water	37.1	63.8	42.4	83	42.4	83	36.1	61.3	36.1	61.3
Total	100	172.2	100	195.8	100	195.8	100	169.7	100	169.7

**Table 6:** Total Dietary Fibre (TDF), Insoluble Dietary Fibre (IDF), and Soluble Dietary Fibre (SDF) displayed as average values ± standard deviation. Different letters in the same row indicate a significant difference at the level of confidence α = 0.05.

	White Wheat Bread (Control)	Fibre-Fortified White Wheat Bread	Vitamin D_3_ + Fibre-Fortified White Wheat Bread	Wholemeal Bread	Vitamin D_3_- Fortified Wholemeal Bread
TDF (g/100 g)	3.81 ± 0.06 ^c^	9.31 ± 0.25 ^b^	10.72 ± 0.31 ^a^	8.54 ± 0.24 ^b^	9.54 ± 0.67 ^ab^
IDF (g/100 g)	2.44 ± 0.03 ^b^	7.30 ± 0.26 ^a^	8.33 ± 0.05 ^a^	7.18 ± 0.28 ^a^	7.76 ± 0.93 ^a^
SDF (g/100 g)	1.38 ± 0.09 ^c^	2.01 ± 0.05 ^ab^	2.39 ± 0.29 ^a^	1.36 ± 0.05 ^c^	1.79 ± 0.27 ^bc^

**Table 7:** Dough characteristics shown as average values ± standard deviation. PMT = peak maximum time; PV = peak viscosity; FV = final viscosity; BV = breakdown viscosity; DDT = dough development time; C2: protein weakening; C3: starch gelatinisation; C4: starch stability; C5: starch retrogradation; HM = Height Maximum. Different letters in the same row indicate a significant difference at the level of confidence α = 0.05.

	White Wheat Flour (Control)	Fibre-Fortified White Wheat Flour	Vitamin D_3_ + Fibre-Fortified White Wheat Flour	Wholemeal Flour	Vitamin D_3_- Fortified wholemeal Flour
**Gluten network development**					
PMT (s)	53.0 ± 2.0 ^b^	52.3 ± 2.3 ^b^	53.7 ± 2.1 ^b^	104.0 ± 12.5 ^a^	106.7 ± 10.1 ^a^
Maximum Torque (BU)	69.3 ± 2.3 ^a^	61.7 ± 0.6 ^b^	60.3 ± 0.6 ^b^	33.7 ± 4.2 ^c^	29.0 ± 1.7 ^c^
**Pasting properties**					
PV (cP)	1378 ± 10.0 ^a^	956 ± 20.0 ^b^	927 ± 63.0 ^b^	708 ± 13.3 ^d^	821 ± 56.0 ^c^
FV (cP)	1781 ± 11.0 ^a^	1353 ± 23.0 ^b^	1352 ± 40.0 ^b^	1118 ± 34.6 ^c^	1331 ± 97.2 ^b^
BV (cP)	520 ± 28.0 ^a^	291 ± 10.0 ^b^	304 ± 26.0 ^b^	240 ± 10.1 ^c^	263 ± 19.8 ^bc^
DDT (min)	1.4 ± 0.8 ^b^	6.1 ± 0.4 ^a^	6.1 ± 0.2 ^a^	7.8 ± 1.9 ^a^	7.9 ± 2.0 ^a^
C2 (Nm)	0.4 ± 0.0 ^b^	0.3 ± 0.0 ^c^	0.3 ± 0.0 ^c^	0.6 ± 0.0 ^a^	0.6 ± 0.0 ^a^
C3 (Nm)	4.8 ± 0.1 ^a^	1.3 ± 0.0 ^c^	1.2 ± 0.0 ^c^	2.1 ± 0.0 ^b^	2.1 ± 0.0 ^b^
C4 (Nm)	1.9 ± 0.0 ^a^	1.1 ± 0.1 ^c^	1.0 ± 0.0 ^c^	1.5 ± 0.1 ^b^	1.6 ± 0.0 ^b^
C5 (Nm)	4.4 ± 0.1 ^a^	2.0 ± 0.5 ^cd^	1.5 ± 0.0 ^d^	2.5 ± 0.1 ^bc^	2.7 ± 0.0 ^b^
**Fermentation**					
HM (mm)	65.9 ± 2.8 ^a^	56.1 ± 2.7 ^b^	59.2 ± 4.9 ^ab^	30.4 ± 0.8 ^c^	27.9 ± 0.7 ^c^
Total CO_2_ produced (mL)	1958 ± 6.6 ^b^	2151 ± 66.5 ^a^	1966 ± 88.1 ^b^	1733 ± 38.6 ^c^	1732 ± 68.4 ^c^

**Table 8:** Bread characterisation for five bread recipes as average values ± standard deviation.—signifies “not applicable”, n.d. signifies “not detected”. L*: lightness, a*: green/red, b*: blue/yellow. Different letters in the same row indicate a significant difference at the level of confidence α = 0.05.

	White Wheat Bread (Control)	Fibre-Fortified White Wheat Bread	Vitamin D_3_ + Fibre-Fortified White Wheat Bread	Wholemeal Bread	Vitamin D_3_— Fortified Wholemeal Bread
Vitamin D_3_ (μg/100 g bread)	n.d.	n.d.	27.2 ± 7.4	n.d.	28.4 ± 7.8
Specific Volume (mL/g)	4.8 ± 0.1 ^a^	4.2 ± 0.4 ^b^	4.0 ± 0.4 ^b^	2.4 ± 0.1 ^c^	2.1 ± 0.0 ^c^
Water Activity	0.97 ± 0.0 ^a^	0.98 ± 0.0 ^a^	0.98 ± 0.0 ^a^	0.98 ± 0.0 ^a^	0.97 ± 0.0 ^a^
**Crumb structure**					
Number of cells	4407 ± 227 ^a^	4551 ± 311 ^a^	4378 ± 275 ^a^	3660 ± 194 ^b^	3653 ± 299 ^b^
Cells diameter (mm)	2.6 ± 0.2 ^a^	2.4 ± 0.2 ^b^	2.4 ± 0.2 ^b^	1.9 ± 0.1 ^c^	1.8 ± 0.1 ^c^
**Bread Texture**					
Hardness (N)	2.3 ± 0.4 ^d^	3.3 ± 0.9 ^cd^	3.6 ± 1.3 ^cd^	23.5 ± 3.8 ^b^	29.9 ± 3.5 ^a^
Staling rate (%)	3.0 ± 1.0 ^a^	2.6 ± 0.9 ^ab^	2.4 ± 0.7 ^ab^	2.3 ± 0.7 ^b^	1.1 ± 0.4 ^c^
**Colour difference ΔE with control**					
ΔE_Crust_	-	2.9 ± 1.9 ^b^	4.7 ± 1.6 ^ab^	4.8 ± 1.9 ^ab^	5.1 ± 0.9 ^a^
ΔE_Crumb_	-	3.1 ± 1.2 ^b^	2.5 ± 0.2 ^b^	15.9 ± 0.8 ^a^	16.4 ± 0.7 ^a^
L*crumb	64.4 ± 2.7	66.9 ± 1.7	66.6 ± 0.0	51.4 ± 0.3	51.0 ± 0.3
a*crumb	−1.2 ± 0.2	−1.1 ± 0.1	−0.9 ± 0.0	6.3 ± 0.1	6.5 ± 0.1
b*crumb	14.7 ± 1.2	16.5 ± 0.8	16.4 ± 0.9	20.0 ± 0.2	20.1 ± 0.1
L*crust	39.5 ± 4.5	37.4 ± 2.3	38.5 ± 3.5	42.5 ± 2.0	43.2 ± 1.1
a*crust	15.7 ± 1.3	15.3 ± 1.4	16.0 ± 0.8	13.4 ± 0.3	13.8 ± 0.0
b*crust	22.6 ± 4.4	22.9 ± 0.6	22.8 ± 4.7	25.5 ± 1.3	25.7 ± 0.7

## Data Availability

The original contributions presented in this study are included in the article material. Further inquiries can be directed to the corresponding author.

## Abbreviations

The following abbreviations are used in this manuscript:

BF	Baker’s Flour
BV	Breakdown Viscosity
DDT	Dough Development Time
DoE	Design of Experiment
FV	Final Viscosity
HM	Height Maximum
IDF	Insoluble Dietary Fibre
MCT	Medium Chain Triglyceride
PMT	Peak Maximum Time
PV	Peak Viscosity
RSM	Response Surface Methodology
SDF	Soluble Dietary Fibre
SDFP	High-Molecular Weight Soluble Dietary Fibre
SDFS	Low-Molecular Weight Soluble Dietary Fibre
TDF	Total Dietary Fibre
WMF	Wholemeal Flour

## Appendix A

**Table A1:** Formulations for ten pre-optimised breads suggested by Design of Experiment, following Response Surface Methodology (RSM). % values are expressed on a flour + fibre basis.

Sample	BF (%)	Oat Fibre (%)	Cellulose (%)	Pectin (%)	Beta-Glucan (%)	Gluten (%)
Pre-optimised-1	82.8	7.6	3.0	1.4	5.2	4.4
Pre-optimised-2	81.1	6.0	2.6	2.6	7.7	4.8
Pre-optimised-3	82.3	9.8	2.0	5.1	0.8	4.6
Pre-optimised-4	79.6	2.9	5.2	4.8	7.5	5.2
Pre-optimised-5	82.4	1.0	5.7	4.6	6.3	4.3
Pre-optimised-6	82.3	3.1	5.5	4.1	5.0	4.4
Pre-optimised-7	77.6	7.5	4.3	1.5	9.1	4.9
Pre-optimised-8	82.5	6.7	4.8	5.0	1.0	4.6
Pre-optimised-9	81.4	9.5	2.8	2.9	3.4	4.6
Pre-optimised-10	81.2	1.8	5.8	3.6	7.6	4.4
